# The diversity of membrane transporters encoded in bacterial arsenic-resistance operons

**DOI:** 10.7717/peerj.943

**Published:** 2015-05-12

**Authors:** Yiren Yang, Shiyang Wu, Ross McCausland Lilley, Ren Zhang

**Affiliations:** School of Biological Sciences, University of Wollongong, NSW, Australia

**Keywords:** Prokaryotes, Arsenic resistance, Membrane transporters

## Abstract

Transporter-facilitated arsenite extrusion is the major pathway of arsenic resistance within bacteria. So far only two types of membrane-bound transporter proteins, ArsB and ArsY (ACR3), have been well studied, although the arsenic transporters in bacteria display considerable diversity. Utilizing accumulated genome sequence data, we searched arsenic resistance (*ars*) operons in about 2,500 bacterial strains and located over 700 membrane-bound transporters which are encoded in these operons. Sequence analysis revealed at least five distinct transporter families, with ArsY being the most dominant, followed by ArsB, ArsP (a recently reported permease family), Major Facilitator protein Superfamily (MFS) and Major Intrinsic Protein (MIP). In addition, other types of transporters encoded in the *ars* operons were found, but in much lower frequencies. The diversity and evolutionary relationships of these transporters with regard to arsenic resistance will be discussed.

## Introduction

Arsenic (As) is the 53rd most abundant element in the earth’s crust, but is the most ubiquitous environmental toxin and carcinogen (Zhu et al., 2014). Capable of inhibiting protein function and cell metabolism through disturbing disulfide bonds and ATP synthesis, arsenic presents hazards to all forms of life. To survive in arsenic environments, organisms have developed a range of arsenic resistance pathways (Tawfik & Viola, 2011).

Generally, these resistance pathways usually follow the process of lowering arsenic concentrations in the cytoplasm by limiting arsenic uptake or promoting arsenic extrusion, or metabolizing arsenic to less toxic compounds (Zhu et al., 2014). Although reduced arsenic uptake is proven to increase arsenic tolerance (Zhao et al., 2009; Elias et al., 2012), the most commonly reported pathway operates via promoted arsenic extrusion. Arsenic extrusion is facilitated through various membrane-bound transporters (Rosen, 1999; Yang et al., 2012). Most reported arsenic transporters extrude arsenite (AsIII), in either inorganic (iAsIII) or organic (oAsIII) forms. Substrate specificities vary between different arsenic transporters (Drobná et al., 2010; Maciaszczyk-Dziubinska, Wawrzycka & Wysocki, 2012).

In bacteria, most inorganic arsenite transporters identified to date belong to either ArsB or ArsY families. ArsB was first found in *Escherichia coli* plasmid R773 (Hedges & Baumberg, 1973) and ArsY (YqcL) in *Bacillus subtilis* skin element (Sato & Kobayashi, 1998). Since ArsY is homologous to the yeast arsenic transporter ACR3 (Bobrowicz et al., 1997), this group of arsenite transporters have also been assigned into the ACR3 (or Acr3) family (Rosen, 1999).

Both ArsB and ArsY are proton motive force-dependent secondary metalloid/H+ antiporters; however, ArsY exhibits greater specificity for arsenite over antimonite (SbIII) (Rosen, 1999; Maciaszczyk-Dziubinska et al., 2010; Maciaszczyk-Dziubinska, Migocka & Wysocki, 2011; Bhat et al., 2011). ArsB and ArsY can also couple with ArsA (ATPase encoded by *arsA*) to form primary arsenite transporter systems which are much more efficient in extruding the toxic metalloid (Rosen, 1996; Zheng et al., 2013).

Despite the similar biological role of ArsB and ArsY, they have evolved independently as only limited sequence homologies (around 20%–40%) can be found between these two families (Bhat et al., 2011). ArsB has 12 transmembrane domains (TMDs) whilst ArsY has 10 (Rosen, 1999). ArsB was classified into the ion transporter superfamily (Prakash et al., 2003) and ArsY was categorized into the bile/arsenite/riboflavin transporter (BART) superfamily (Mansour et al., 2007). A preliminary study of genome databases revealed a wide distribution of ArsY homologues in bacteria, archaea and eukaryotes (mainly fungi and some lower plants), while fewer ArsB homologues can be found outside the bacteria kingdom (Yang et al., 2013, unpublished data). Thus, the current explanation for the divergence between ArsB and ArsY is convergent evolution (Mukhopadhyay et al., 2002; Galperin, Walker & Koonin, 1998).

While ArsB and ArsY have been recognized as the dominant arsenite transporters in bacteria, there have been a few reports of other possible bacterial arsenic transporters. For example, a bi-directional arsenite-transporting protein, Aqps, has been identified in *Sinorhizobium meliloti* (Yang et al., 2005). A permease protein (ArsP) encoded by the arsenic resistance (*ars*) operon of *Campylobacter jejuni* was reported as an organic arsenic transporter (Wang et al., 2009; Shen et al., 2014). Two other putative transporters belonging to the major facilitator superfamily (MFS) have also been identified as the assumed products of bacterial *ars* operons (Chauhan et al., 2009; Drewniak et al., 2013). These indicate that, in addition to ArsB and ArsY, other membrane transporters have been recruited to deal with arsenic toxins in some bacteria. However, there is a lack of systematic study of these transporters in terms of their diversity and occurrence.

Utilizing the rapidly accumulated sequence data of full bacterial genomes, we searched about 2,500 genomes and analyzed over 700 putative membrane transporters encoded in the identified *ars* operons. Our findings indicate that bacteria employ more diverse membrane transporters than previously known in combating arsenic toxicity.

## Materials and Methods

### *ars* operon data collection and verification

The sequence of *Bacillus sp.* CDB3 *ars* cluster 1 (Bhat et al., 2011) was used for mining putative *ars* operon data using the tblastn program and searching the NCBI genome database (http://www.ncbi.nlm.nih.gov/sutils/genom_table.cgi). Putative *ars* operons (updated to October 2013) were then verified by cross-checking in the ProOpDB (predicted) operon database (http://operons.ibt.unam.mx/OperonPredictor/) (Taboada et al., 2012). Data from some literature-reported functional *ars* operons, whose host full-genome sequences are not available, were also used in this study.

### Classification of transporter proteins

All membrane transporter-like sequences were extracted from the operon database based on their homologies to other known transporters. The PSIPRED Protein Sequence Analysis Workbench (http://bioinf.cs.ucl.ac.uk/psipred/) and TMpred (Hofmann & Stoffel, 1993) were used to verify the transmembrane span of extracted sequences.

These were then classified into putative family groups based on BLAST results in the Transporter Classification (TC) system (http://www.tcdb.org/) and the NCBI protein database. The HHpred protein prediction tool (http://toolkit.tuebingen.mpg.de/hhpred) was applied when BLAST could not provide sufficient information for such family classification. WebLogo version 2.8.2 (Crooks et al., 2004) was used to further identify conserved regions within different protein families.

### Phylogeny analysis

The MAFFT multiple sequence alignment program was used for sequence alignment (http://mafft.cbrc.jp/alignment/software/) and a rough phylogeny tree was generated using UPGMA (Unweighted Pair Group Method with Arithmetic Mean) algorithms (Sneath & Sokal, 1973). Neighbor-joining trees were applied in further analysis of separated transporter families.

## Results and Discussion

### Classification of membrane transporters encoded in *ars* operons

Of about 2500 bacterial genomes surveyed (full-genome data published before October 2013), 685 *ars* operons were identified and 717 putative membrane transporters were extracted. In addition, a number of experimentally characterized arsenic transporters were included in our study, including ArsB (Chen et al., 1986; Bröer et al., 1993; Bruhn et al., 1996; Ryan & Colleran, 2002), ArsY (ACR3) (Hu et al., 2011; Bhat et al., 2011), Aqps (Yang et al., 2005) and GlpF (Meng, Liu & Rosen, 2004). The large majority of these membrane proteins are significantly homologous to known arsenic transporters (Table 1) with the most abundant being ArsY (484; 66.5%) followed by ArsB (111; 15.2%), confirming previous findings. There were a large number of putative *ars* proteins annotated as AR (arsenic resistance) in the genome database due to inadequate identification. These were found to be either ArsY (mostly) or ArsB proteins and are reclassified in this study. Three other types of transporters were also observed to occur quite frequently, being the 49 homologues of the novel permease ArsP (Wang et al., 2009; Shen et al., 2014), 35 candidates of Major Facilitator protein Superfamily (MFS) and 18 putative Major Intrinsic Proteins (MIPs). In addition, 20 other putative membrane transporter proteins were also found, including 5 ATP-Binding Cassette transporters (Table 1).

**Table 1:** Membrane transporters encoded in *ars* operons.

Transporter family	Occurrence
ArsY (ACR3)	484
ArsB	111
ArsP	49
Major Facilitator Transporter (MFS)	35
Major Intrinsic Protein (MIP)	18
ABC Transporter (ABC)	5
Chromate Transporter (CHR)	2
Co/Zn/Cd Cation Transporter Protein (CDF)	2
The Drug/Metabolite Transporter (DMT)	2
NhaP type Na + (K + )/H + Antiporter (CPA)	1
Hypothetical Membrane Transporter	8

**Figure 1:**
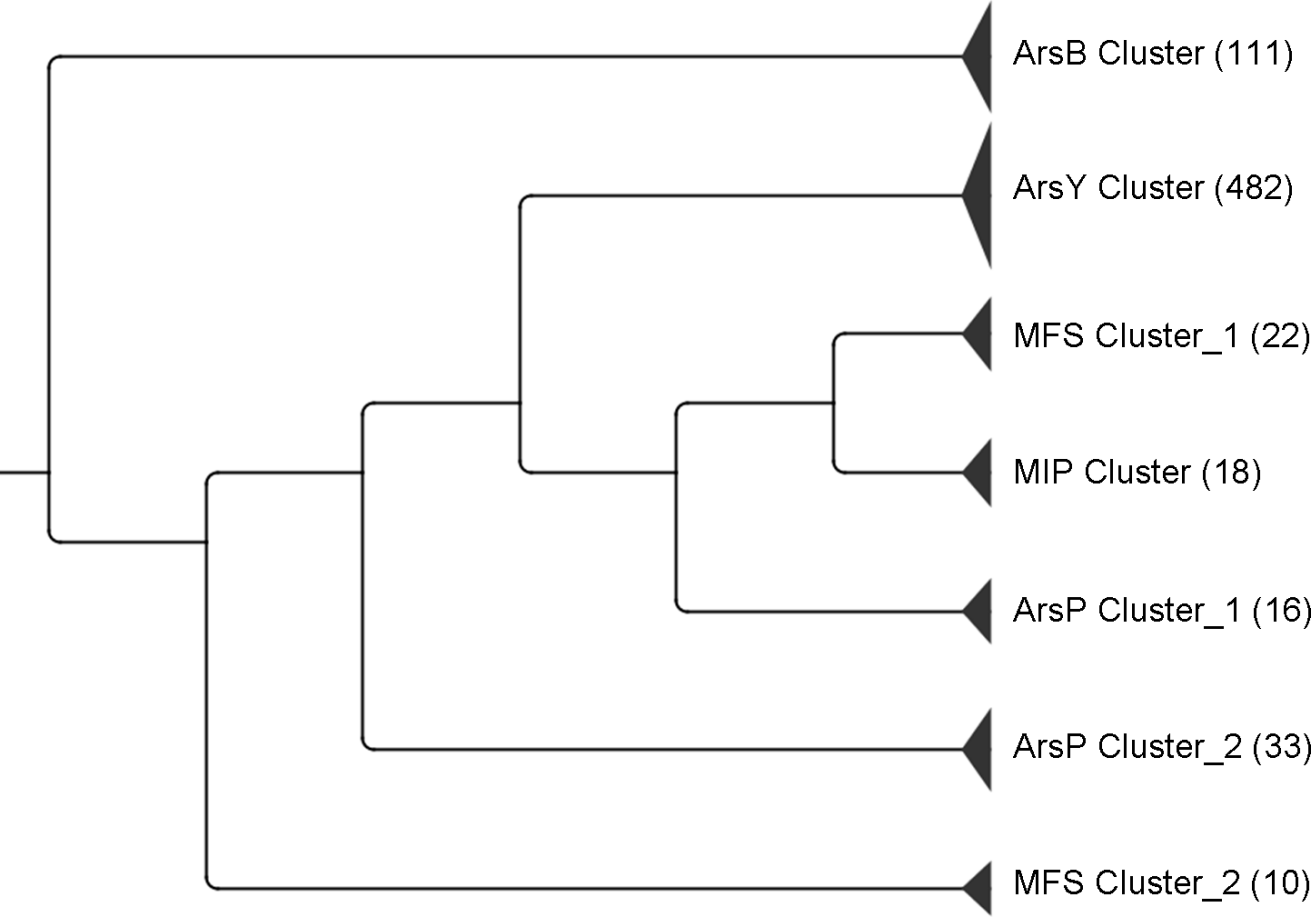
UPGMA tree of the five major transporters encoded in *ars* operons. The numbers in brackets represent the number of proteins that clustered together. Two ArsY and three MFS homologous sequences were not included as they were not clustered closely with their respective families.

An UPGMA tree was constructed using data from the five major protein families (Table 1). Distinct clusters of ArsBs, ArsYs and MIPs were found whilst ArsP and MFS proteins were divided into two sub-clusters (Fig. 1). Two putative ArsY and three MFS homologues were not clustered closely with their respective families and thus were not included in this tree.

### Major facilitator protein superfamily

**Figure 2:**
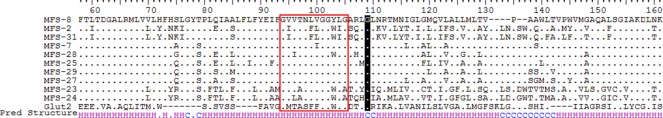
Partial sequence alignment of human liver Glut2 and MFS sub-cluster_2 (Fig. 1), along with secondary structure prediction (HHpred). Dots represent identical amino acid residues. There is 19% identity and 36% similarity of amino acids in this region of aligned sequences. Sequences of putative glycine zipper structures are within the box, followed by a putative functional glycine (shadowed). Bottom line of Pred Structure: H, helix structure; C, coil or loop structure.

The MFS proteins represent a large family of secondary transporters carrying small solutes (Pao et al., 1998). To date at least 74 sub-families have been classified, each of which transports a specific substrate (Reddy et al., 2012), although no arsenic-specific sub-family has been nominated. A human MFS member, the liver sugar porter Glut2, was previously proposed as a bi-directional arsenic transporter (Drobná et al., 2010). Only two MFS proteins have been reported encoded in bacterial *ars* clusters (Chauhan et al., 2009; Drewniak et al., 2013); however, no experimental evidence yet demonstrates their involvement in arsenic resistance. A total of 35 putative MFS proteins were identified in this study, accounting for nearly 5% of the membrane transporters encoded by the surveyed *ars* operons. In the phylogeny tree they were divided into at least two sub-clusters (Fig. 1), and proteins from MFS sub-cluster 2 showed greater conservation compared with MFS sub-cluster 1 (data not shown). Certain homologies were also found between MFS sub-cluster 2 proteins and the N-terminal half (residues 50–250) of human liver protein, Glut2 (Fig. 2). Multiple conserved glycines detected within transmembrane helix regions were suggested as key structures of these proteins, for their motif homologies with previously reported GXXXGXXXG glycine zippers (Kim et al., 2005). Moreover, following the proposed glycine zipper structure, an additional highly conserved glycine was located in the coil region between two TMDs, suggesting a possible substrate-binding site for these MFS proteins. Assumingly, these *ars* operon-encoded MFS proteins function in their host bacteria against arsenic stress, but experimental proof is required. Whether the two groups of MFS transporters have different substrate specificities also warrants investigation.

### Major intrinsic proteins

**Figure 3:**
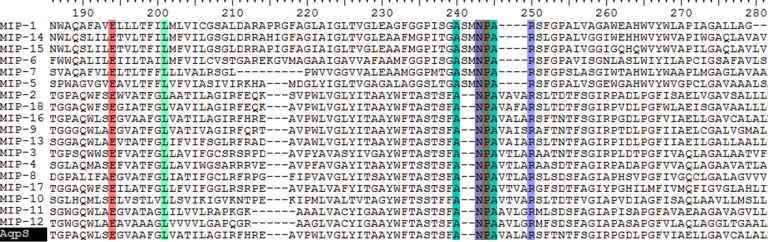
Partial sequence alignment of MIPs encoded by the *ars* operons with Aqps from *Sinorhizobium meliloti* SM1021 indicated. Identical residues including MIP signature motif NPA are highlighted.

Composed of aquaporins (AQPs) and glycerol-permeable aquaglyceroporins (GLPs), MIPs are water channels, some of which can facilitate the efflux and intake of arsenite (Bienert, Schüssler & Jahn, 2008). Arsenite-transporting AQPs have been identified in numerous eukaryotes including plants, yeasts, fish, mice and humans (Bienert et al., 2008; Maciaszczyk-Dziubinska, Wawrzycka & Wysocki, 2012; Yang et al., 2012). Apart from arsenite, some of the AQP transporters in plants and animals are also permeable to methylated arsenicals (Maciaszczyk-Dziubinska, Wawrzycka & Wysocki, 2012). In bacteria, two MIPs have been proved capable of arsenite transport: Aqps in *Sinorhizobium meliloti* (Yang et al., 2005) and GlpF in *E. coli* (Meng, Liu & Rosen, 2004). Sequence alignments of the 18 putative MIPs recognized in this study revealed two highly conserved NPA motifs confirming the transportation role of these MIPs (Fig. 3). Previous studies attributed their arsenic transportation capability to molecular similarity between As(OH)_3_ and glycerol (Yang et al., 2005; Mukhopadhyay, Bhattacharjee & Rosen, in press). It is assumed that all of the 18 *ars* operon-encoded MIPs serve the same arsenic transportation role as exemplified by the *Sinorhizobium* Aqps (Yang et al., 2005).

### ArsP permeases

Forty-nine of the putative membrane proteins showed good homology to the ArsP protein, a novel permease encoded by an *ars* operon in *Campylobacter jejuni*, which has been recently proved as an organic arsenic (roxarsone and nitarsone) transporter (Shen et al., 2014). These ArsP homologues were found amongst 11 bacterial phyla, indicating a wide occurrence of this permease in bacteria that tolerate arsenic toxins, particularly the organic form. Furthermore, homologous proteins were also found widely in archaea with a few in eukaryotes (Yang et al., 2013, unpublished data) suggesting an ancient origin.

**Figure 4:**
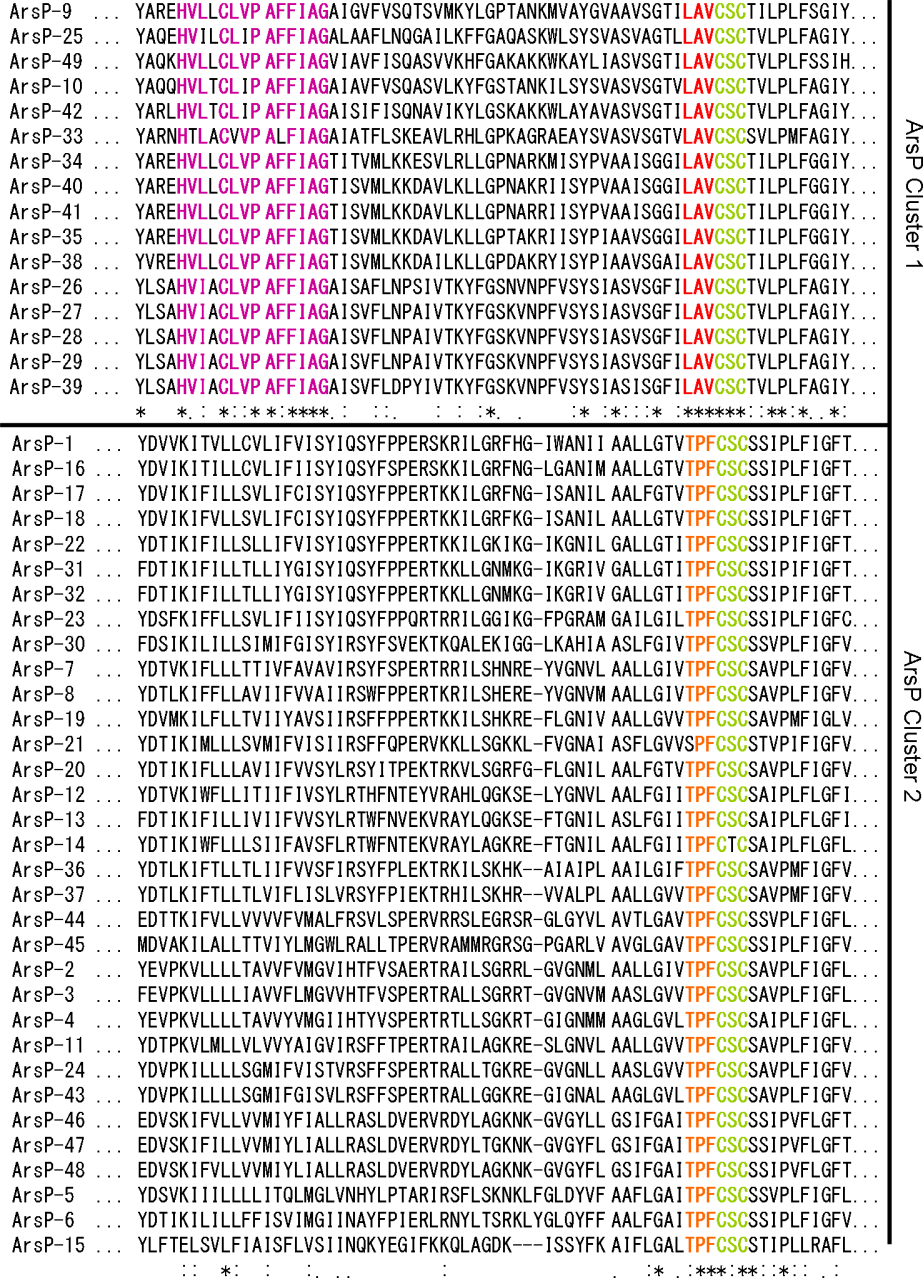
Partial sequence alignments of ArsP cluster 1 and cluster 2 (Fig. 1). The conserved motifs are highlighted.

All of the surveyed ArsP homologues have a conserved C(S/T)C motif located on the transmembrane domain (Fig. 4), The well-conserved C(S/T)C motif in these ArsP proteins may assume an important role in the transporter function as suggested by Shen et al. (2014). Many studies have indicated the significant role of conserved cysteine residues in arsenic resistance-associated proteins including ArsA, ArsR, ArsC and ArsD (Rosen, 1999; Bhat et al., 2011). A highly conserved cysteine has also been identified in the pore helix of ArsY, although its biological role remains unknown (Fu et al., 2009).

Unsurprisingly, given the divergence within ArsPs noted in Fig. 1, at least two ArsP clusters were distinguished. Based on the ClustlW (Fig. 4) and WebLogo (Fig. S1) analyses, three distinctive sequence differences between the two clusters were observed. The cluster 1 proteins contain a conserved motif of Hv (L/I)XClv PAf FIAG towards their N-termini that is absent from cluster 2. In addition, directly upstream of the universal C(S/T)C motif, LAV is conserved in cluster 1 compared with t PF in cluster 2. It is also interesting to note the conservation of another cysteine residue in the N-terminal region among cluster 1 ArsPs. Whether this additional conserved cysteine and the associated motifs relate to different substrate specificity or affinity has also yet to be investigated.

**Figure 5:**
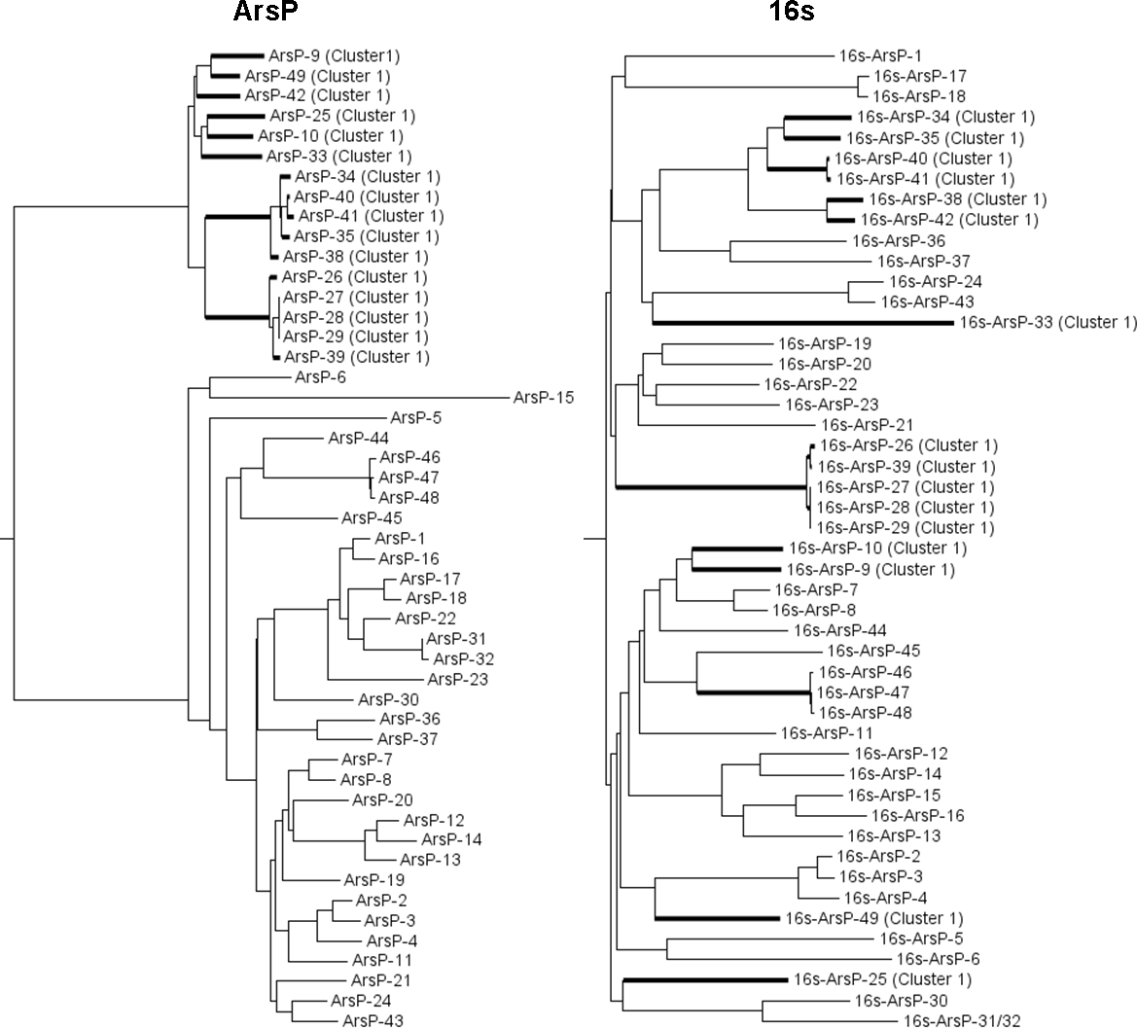
Comparison of neighbor joining trees of the 16S rDNA and ArsP clusters. The members of ArsP cluster 1 are highlighted.

The phylogeny tree of ArsP sequences does not match that of 16S rDNA sequences (Fig. 5), indicating that the divergence between two ArsP clusters was not due to division of bacterial species and thus may be function-related.

### Minor groups of transporters

For the other 20 putative transmembrane proteins recognized (Table 1), only the five ABC transporters appear likely to have arsenic-transporter functions. ABC transporters facilitating arsenic extrusion have been found in several eukaryotes (Cole et al., 1994; Kala et al., 2000; Xie et al., 2004; Song et al., 2010; Wawrzycka et al., 2010; Wysocki et al., 2001; Mukhopadhyay et al., 2002; Tan et al., 2014). ABC transporters normally contain 12 transmembrane regions and are one of the biggest membrane superfamilies across all domains of life (Johnson, Lewinson & Rees, 2009). The fact that less than 1% of the membrane transporter proteins surveyed in this study that were ABC transporters suggests a less important role played by this type of transporter in bacterial arsenic resistance. These bacterial transporters may also carry arsenic non-specifically as do their counterparts in eukaryotes (Song et al., 2010; Xie et al., 2004; Kala et al., 2000; Wysocki et al., 2001; Wawrzycka et al., 2010).

The other membrane proteins in Table 1 may or may not relate directly to arsenic transportation. CHR family proteins are known to confer chromate resistance (Díaz-Magaña et al., 2009); CDF family proteins are known to transport a range of heavy metals (Co, Zn and Cd) but not arsenic (Paulsen & Saier, 1997) and the DMT family is involved in export of a wide range of drugs and metabolites (Jack, Yang & Saier, 2001). The *ars* operon-encoded protein homologues may have retained the same functions or diverged with arsenic-carrying ability. The presence of these transporter genes within *ars* operons might have resulted from survival pressures or random genomic translocation events.

### Co-existence of the membrane transporters

While ArsY and ArsB were found to be dominant in the *ars* operon-encoded membrane transporters (Table 1), it was also observed that there is no case of co-existence of ArsB and ArsY in a single operon. There have been a few reported cases of their co-existence with other transporters (Chauhan et al., 2009; Wang et al., 2009). This study revealed that out of the 122 other transporters identified, 84 (68.9%) are located in operons which encode either ArsY (89.3%) or ArsB (10.7%) (Table 2). All of the 18 MIPs were found standalone; that is, none of the *ars* operons bearing MIPs encode another membrane transporter, which further supports the proposition that these MIPs play an arsenic transportation role. It was interesting to note that around 40% of surveyed ArsP (including members of both sub-clusters) are also encoded alone. All the other types of membrane transporters identified in this study were encoded together with either ArsB or ArsY in a particular *ars* operon.

**Table 2:** Co-existence of various membrane transporters encoded by *ars* operons.

	With ArsB	With ArsY	Encoded alone
ArsP	5	24	20
MFS	2	33	
MIP	0	0	18
ABC	0	5	
CHR	0	2	
CDF	0	2	
DMT	1	1	
CPA	0	1	
Other	1	7	
Total	9	75	38

The reason for the co-existence of two membrane transporters may be explained by their specificities to carry different arsenicals. It has been reported that two such transporters, ArsY and ArsP encoded by the four-gene *C. jejuni ars* operon (Wang et al., 2009; Shen et al., 2014), coordinate to transport inorganic and organic arsenic toxins, respectively. Thus, the co-existence of two or more membrane transporter genes in a single *ars* operon may have evolved as a defense mechanism due to increased levels and chemical complexity of arsenicals in a certain environment.

## Conclusions

The transporters encoded in bacterial *ars* operons form a more diverse group than previously thought, suggesting that a variety of membrane transporter proteins are employed for arsenic tolerance. Arsenic toxicity has been affecting all living organisms since probably the origin of life and the membrane transporter-based cellular extruding system most likely originated early in evolution. ArsY and ArsB are the dominant, and probably most ancient, types of arsenic transporters in bacteria. The minor types may serve either complementary function, with different substrate specificities in certain environments, or as substitutes derived from other types of transporters where ArsB or ArsY are absent. Much still remains to be learned about these important proteins and bacteria have provided an excellent system for such investigations.

## Supplemental Information

10.7717/peerj.943/supp-1 Figure S1 WebLogo alignment of ArsP cluster1 and cluster 2 Distinct conserved region for each cluster were noted.
