# Supplementary figures and images for: The diversity of membrane transporters encoded in bacterial arsenic-resistance operons
Source: PeerJ. 2015 May 12;3:e943. doi: 10.7717/peerj.943 (PMC4435449; doi:10.7717/peerj.943)

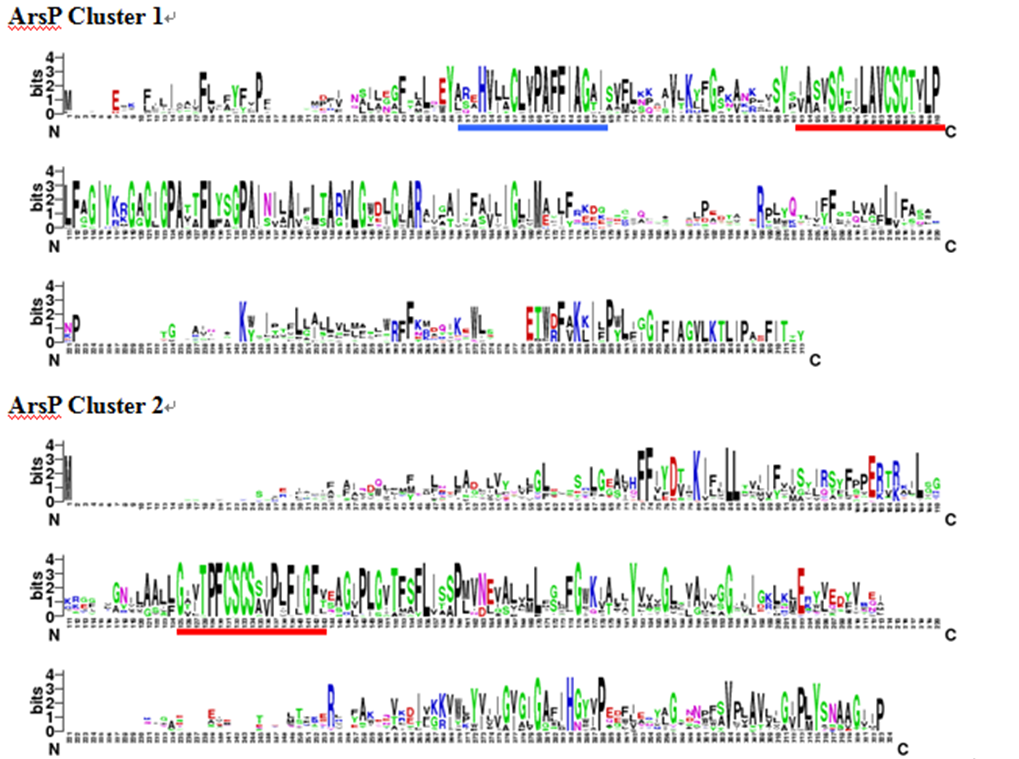

Supplement: Figure S1 — Distinct conserved region for each cluster were noted. [file peerj-03-943-s001.png]
